# Feline Oncogenomics: What Do We Know about the Genetics of Cancer in Domestic Cats?

**DOI:** 10.3390/vetsci9100547

**Published:** 2022-10-04

**Authors:** Latasha Ludwig, Melanie Dobromylskyj, Geoffrey A. Wood, Louise van der Weyden

**Affiliations:** 1 Department of Pathobiology, Ontario Veterinary College, University of Guelph, Guelph, ON N1G 2W1, Canada; 2 Finn Pathologists, Histopathology Department, Diss, Norfolk IP21 5TT, UK; 3 Wellcome Sanger Institute, Wellcome Genome Campus, Hinxton, Cambridge CB10 1SA, UK

**Keywords:** cat, feline, cancer, oncology, genes, genetic, genome, oncogenetics, cytogenetics, methylation

## Abstract

**Simple Summary:**

Cancer is a significant cause of suffering and death in domestic cats. In humans, an understanding of the genetics of different types of cancers has become clinically important for all aspects of patient care and forms the basis for most emerging diagnostics and therapies. The field of ‘oncogenomics’ characterises the alterations of cancer-associated genes that are found in tumours. Such a thorough understanding of the oncogenome of human tumours has only been possible due to a high-quality reference genome and an understanding of the genetic variation that can exist between people. Although a high-quality reference genome for cats has only recently been generated, investigations into understanding the genetics of feline cancers have been underway for many years, using a range of different technologies. This review summarises what is currently known of the genetics of both common and rare types of cancer in domestic cats. Drawing attention to our current understanding of the feline oncogenome will hopefully bring this topic into focus and serve as a springboard for more much-needed research into the genetics of cancer in domestic cats.

**Abstract:**

Cancer is a significant cause of morbidity and mortality in domestic cats. In humans, an understanding of the oncogenome of different cancer types has proven critical and is deeply interwoven into all aspects of patient care, including diagnostics, prognostics and treatments through the application of targeted therapies. Investigations into understanding the genetics of feline cancers started with cytogenetics and was then expanded to studies at a gene-specific level, looking for mutations and expression level changes of genes that are commonly mutated in human cancers. Methylation studies have also been performed and together with a recently generated high-quality reference genome for cats, next-generation sequencing studies are starting to deliver results. This review summarises what is currently known of the genetics of both common and rare cancer types in cats, including lymphomas, mammary tumours, squamous cell carcinomas, soft tissue tumours, mast cell tumours, haemangiosarcomas, pulmonary carcinomas, pancreatic carcinomas and osteosarcomas. Shining a spotlight on our current understanding of the feline oncogenome will hopefully serve as a springboard for more much-needed research into the genetics of cancer in domestic cats.

## 1. Introduction

**Figure 1:**
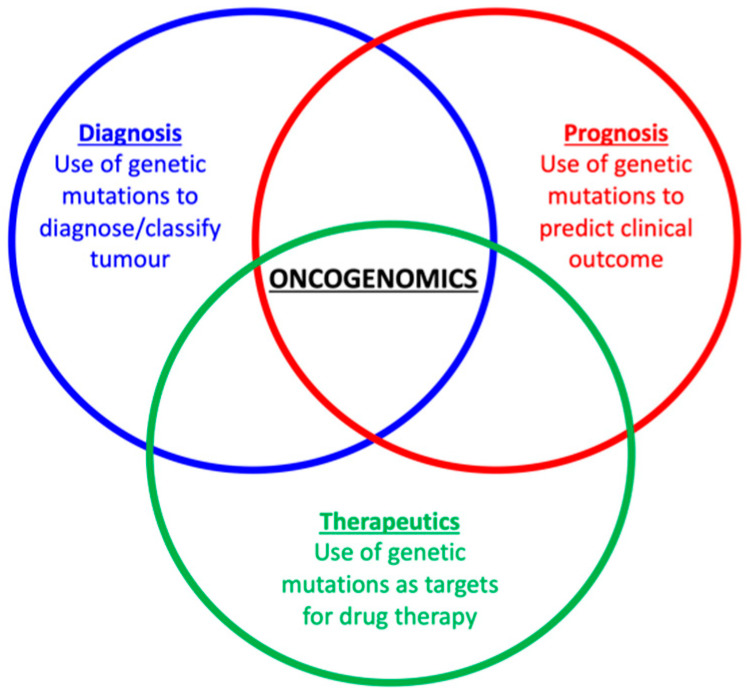
Potential benefits that can come from understanding the feline oncogenome, such as the development of diagnostic tools, prognostic markers and/or targeted therapies.

Between 25–56% of households in the UK, USA and Canada report having a pet cat [1,2,3]. Cancer is a significant cause of morbidity and mortality in domestic cats with many studies showing that the majority of tumours arising in these animals are malignant (Table 1). Sadly, however, feline cancers have not been studied as extensively as human or canine cancers. In particular, there have been comparatively fewer investigations into the genetics of these tumours and as such there are missed opportunities; once the oncogenome of feline cancers have been more fully characterised, there is much potential for the development of novel diagnostic tools, prognostic markers and/or more targeted therapies (Figure 1). In addition, there are benefits in understanding the aetiology of these tumours, such as detecting the presence of viruses or a mutational signature demonstrating exposure to carcinogens such as ultraviolet (UV) radiation or tobacco smoke. Finally, analysis of the germline of tumour-bearing cats will potentially allow identification of possible tumour-predisposing alleles, which would allow such genes to be bred out of the population. Thus, a thorough understanding of the oncogenome of feline cancers is critical.

As detailed in the sections below, many early studies investigating the genetics of feline cancers have used cytogenetics, which looks at the structure of chromosomes for changes such as broken, missing, rearranged or extra chromosomes. Other studies have looked at DNA methylation patterns, as methylation is an epigenetic mechanism of controlling gene expression without altering the DNA sequence. Those that have performed genetic studies to investigate the mutation or expression status of genes have mostly taken a single gene approach, examining the mutational status of specific exons of a particular gene known to be involved in human cancer, with the most commonly investigated gene being the tumour suppressor gene, *TP53*. *TP53* encodes p53 which governs a complex anti-proliferative transcriptional program critical for counteracting transformation and tumour growth and is the most mutated gene in human cancers [10]. For a more genome-wide approach, a well-annotated genome is required.

In 2006, the International Cat Genome Sequencing Consortium (ICGSC) released ‘ASM18133v3’ as the first assembly of the domestic cat (*Felis catus*) genome reference. It was generated from the DNA of a female Abyssinian breed cat (named ‘Cinnamon’) kept by Dr. Kristina Narfstrom at the University of Missouri. This 2× coverage draft assembly was highly fragmented, requiring 174,000 contigs to cover half the cat genome, and both the genome assembly and annotation were heavily dependent upon comparative mapping to human and dog genomes [11]. The ICGSC has gradually improved this reference genome over the years with many version releases benefitting from advances in sequencing, assembly and mapping technologies. In 2014, an ~14× coverage whole-genome assembly for ‘Cinnamon’ was released, closing gaps of sequence that were unavoidable with previous low-coverage shotgun genome sequencing; it spanned 2.35 Gb and included annotation of 21,865 protein-coding genes [12,13]. The current assembly version, ‘Felis_catus_9.0’ was released in 2017 and has exceptionally long gap-free segments and improvements in genomic features include defining pseudogenes, small non-coding RNAs, lincRNAs and novel genes that were absent in previous versions of Felis_catus [14]. This is now a high-quality reference genome for the domestic cat.

The present paper summarises the genetic investigations of feline cancers that have been carried out to-date, utilising the range of technologies that have been available over the years.

## 2. Lymphoma

**Figure 2:**
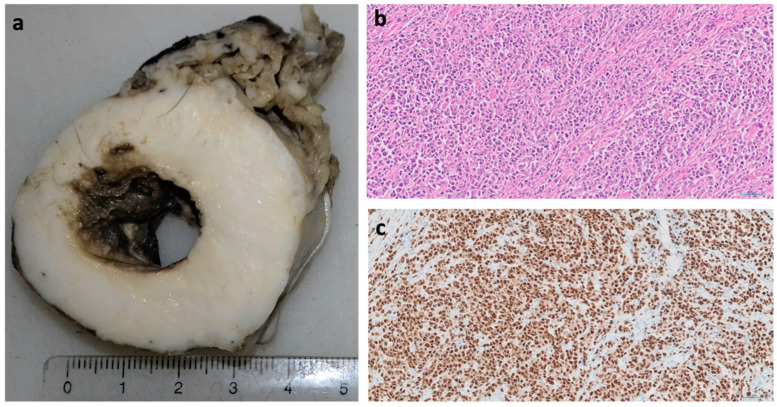
A case of a gastrointestinal B-cell lymphoma in a cat. An 8-year old female Domestic short hair cat presenting with an abdominal mass underwent surgery to remove the lesion, which was sent for histopathologic examination. (**a**) Macroscopic image of the excised mass (the area in white is the tumour). (**b**) Microscopic image of a representative section of the mass showing neoplastic cells arranged in sheets or infiltrating between and sometimes separating pre-existing tissue structure (HE-stained, ×20 magnification). (**c**) Immunohistochemistry image of the lymphoma showing positivity for Pax5, confirming it is of B-cell origin (×20 magnification).

Lymphoma is a malignant cancer of lymphocytes, and thus many different tissues can be affected, with common sites including the gastrointestinal tract (GI; Figure 2), mediastinum (organs in the chest, such as lymph nodes and thymus) and kidneys. GI lymphoma is the most common type, accounting for 50–70% of the cases [15]. Mediastinal and renal lymphoma are frequently associated with feline leukaemia virus (FeLV) and as cats have become more routinely vaccinated for this virus, these two sites of lymphoma are now less common [16], although this can differ between countries [17]. Despite this decline in viral-associated cases in some countries, studies have shown that GI lymphoma is becoming more prevalent [18].

Lymphoma is usually treated with chemotherapy, and/or surgery depending on the location, and the prognosis typically depends on the location and histologic grade of the tumour. Most cases of gastrointestinal lymphoma are low-grade and with standard treatment of chlorambucil and glucocorticoids up to 96% of cats may see a clinical response (complete or partial remission) and this may last for 2–3 years if complete remission is achieved [19,20], whereas only 30% of cats with high-grade GI lymphoma have achieved complete remission using a standard multi-agent chemotherapy protocol, with a median progression-free interval of only 50 days [21]. The reported median survival time (MST) for mediastinal lymphoma is variable and may be dependent on FeLV status; however, recent literature generally only includes a small number of FeLV-positive cases [22,23,24]. Feline renal lymphomas have a worse prognosis (MST of up to 203 days) compared to other locations, regardless of treatment with either glucocorticoids alone or in combination with multiagent chemotherapy [25].

There have only been limited investigations into understanding the genetics of feline lymphoma. Several of these have involved the use of feline lymphoma cell lines (Table 2) and there are only a handful that have involved the use of lymphoma tissues from cats.

Investigation of lymphoma tissue samples found that *TP53* was mutated at codon 282 (exon 8) in a case of feline lymphoma [32] and at codon 199 in 1/8 cases of feline lymphoma [26]. In addition, southern-blot analyses confirmed there was no deletion or rearrangement of either the *p21WAF1* or *p27Kip1* genes in 19 cases of feline leukaemia and lymphoma, with the analysis of *p27Kip1* cDNA in 10 of these cases indicating there were no non-synonymous mutations present [33]. Investigations into *N-RAS* found a somatic non-synonymous mutation giving rise to a Q61K amino acid change in 1/15 feline lymphomas (Q61K is a hot-spot mutation in human cancers) [34]. Investigations into Fas, which belongs to the tumour necrosis factor receptor family and transduces the death signal after binding to the Fas ligand, led to one group detecting *FAS* mRNA in 7/11 feline lymphoma samples, with some samples showing the presence of alternatively spliced transcripts (similar to what they had found using feline lymphoma cell lines) [35]. More recently, analysis of 42 feline T-cell GI lymphoma samples by immunohistochemistry demonstrated broad activation of STAT3 and STAT5B, and screening for known activating mutations in the same samples identified the presence of the *STAT5B^N642H^* driver mutation (found in humans with type II enteropathy-associated T-cell lymphoma [36]) in 7/42 (17%) samples, with the majority being found in the low grade subtype (known as enteropathy-associated T-cell lymphoma type II or small cell lymphoma), which is the most common subtype [37].

## 3. Mammary Tumours

**Figure 3:**
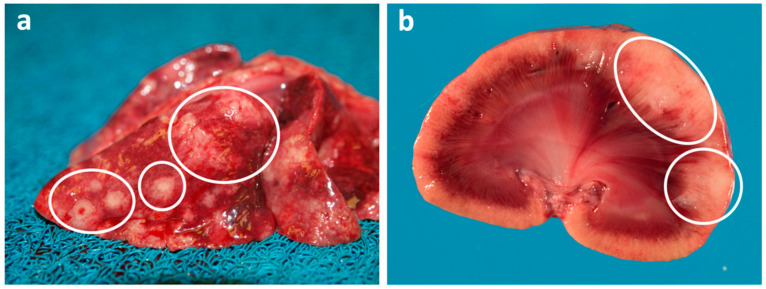
**Macroscopic images of a metastatic mammary carcinoma in a cat.** Postmortem examination of a 6-year old female Domestic long hair cat with a mammary carcinoma that had metastasized to the (**a**) lung and (**b**) kidney. White circles show the tumours in each organ. Photos courtesy of Dr. Jeff Caswell (Department of Pathobiology, University of Guelph).

Mammary tumours are the third most frequently occurring tumour in cats. The vast majority of feline mammary tumours are aggressive and malignant (~85%), with multiple tumours and metastasis frequently seen at time of diagnosis, and relapse with rapid progression generally resulting in a poor prognosis (Figure 3) [38]. Most of the malignant feline mammary tumours are classified as carcinomas, which are divided into different histological subtypes [39]. Surgery is the most widely used treatment for mammary neoplasms in the cat (complete unilateral or bilateral mastectomy to reduce chance of local recurrence), either alone or in combination with chemotherapy (although the overall benefit of adjuvant chemotherapy remains unproven [40]).

As the survival times (disease-free survival (DFS) and cancer-specific overall survival (OS)) of cats with mammary carcinoma are short, prognostication is essential. Molecular classification of feline mammary carcinomas using five markers (ER, PR, HER2, Ki-67 and CK 5/6) has been shown to be prognostic, similar to that seen in human breast cancer patients [41]. For example, several studies have found that the Luminal A subtype (ER^+^ and/or PR^+^, HER2^−^, Ki-67^low index^) is associated with the highest OS whereas the triple negative basal-like subtype (ER^−^, PR^−^, HER2^−^, CK5/6^+^) is associated with the lowest OS [41,42,43]. Analysis of copy number variation (CNV) in 33 female cats with tubulopapillary and solid mammary carcinoma followed for 2 years post-surgery revealed important survival differences between molecular subtypes (but not histopathological subtypes); luminal A tumours exhibited the highest DFS/OS and were associated with the lowest amount of CNVs, whereas the basal-like triple negative mammary carcinomas had the worst DFS/OS and were the most aberrant [43]. There was an association between specific CNVs and poor prognosis; copy-number losses in chromosome B1 (1–23 Mb) and copy-number gains in B4 (1–29 Mb) and F2 (64–82.3 Mb) were associated with poor outcomes [43]. Interestingly, several potential prognostic markers identified in these feline mammary carcinomas were recently found to be relevant prognostic markers in human triple negative basal-like breast cancer [44].

Several studies have looked at the expression of genes known to be important in breast cancer in humans, such the tyrosine kinase receptor proto-oncogene *RON*, and the *ERBB2* proto-oncogene (also known as HER2, neu), to determine if they also show altered expression in feline mammary tumours. The short form isoform of RON (*sf-RON*) and *ERBB2* are overexpressed in human breast cancer and correlate with poor prognosis [45,46]. In feline mammary carcinomas, *sf-RON* mRNA expression was found in 18/47 (38%) cases, and was associated with poorly differentiated tumours, a shorter disease-free interval and a shorter overall survival [47]. *ERBB2* mRNA levels have been found to be elevated in 44–54% of mammary carcinomas in cats (6/11 cases [48] and 12/44 cases [49]), although numerous studies have shown that the majority of these tumours do not carry *ERBB2* gene amplifications [49,50,51]. One study found a significant association between *ERBB2* RNA levels and tumour malignancy as well as molecular subtypes; higher *ERBB2* expression correlated with lower malignancy grade and with the luminal A and HER2 subtypes, whereas the lowest *ERBB2* levels correlated with the triple negative tumours [49]. Another study found that relative to disease-free tissue from the same cat, mammary carcinomas showed overexpression of several other cancer-associated genes, including *CCND1*, *PKM2*, *PTBP1* and *TOP2α*, whilst levels of *TP53*, *c-MYC*, *YBX1* and *FUS* remained unchanged [52]. Interestingly, positive correlations were found between tumour size and *YBX1* RNA levels, lymph node metastasis and *c-MYC* RNA levels [52]. A study of *TWIST1* mRNA expression in 25 feline mammary gland tissues (7 normal glands, 3 hyperplastic lesions, 1 benign tumour and 14 carcinomas) found that the carcinomas had significantly lower *TWIST1* mRNA levels than benign lesions and disease-free mammary glands [53].

Genomic sequence variants in feline mammary tumours have not been widely investigated and to-date only single genes have been looked at, specifically genes known to be mutated in human breast cancer. Somatic alterations in *TP53* in feline mammary tumours have been reported in individual cats, including a missense mutation in exon 8 of a solid mammary carcinoma [54], a deletion of a tandem repeat (involving codons 251–256) of exon 7 in a tubulopapillary mammary carcinoma [55] and a missense mutation in exon 5 of a mammary adenocarcinoma [56]. Similarly, there was report of a cat with mammary carcinoma carrying a pathogenic somatic mutation in exon 11 of *BRCA2* [57]. One study looked at the coding region of the *TWIST1* gene in 34 feline mammary carcinomas, but found no somatic variants present [53].

There have been very few genetic studies on pre-disposition to mammary tumours in cats, with the focus having been to look for the presence of germline mutations in *BRCA1* and *BRCA2*, as the cumulative breast cancer risk in humans is 72% for *BRCA1* and 69% for *BRCA2* carriers [58]. One study found that none of the 24 cats with mammary carcinoma they analysed showed any variants in these genes [59], though a more recent study found that 3/9 cats with mammary carcinoma carried germline variants in exon 9 of *BRCA1* and they postulated that these might be associated with a higher risk of hereditary mammary carcinogenesis [57]. In humans, germline mutations in the *TWIST1* oncogene have been suggested to predispose to breast cancer [60], however, a study of 34 feline mammary carcinomas only identified two intronic germline variants (535delG and 460C >T, in 3 and 4 cases, respectively) and noted there was no association between these alterations and *TWIST1* mRNA levels [55].

## 4. Squamous Cell Carcinoma

**Figure 4:**
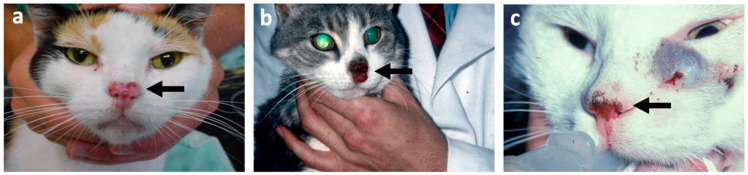
Three cases of cutaneous squamous cell carcinoma (SCC) on the nasal planum of cats. (**a**) Macroscopic image of an early cutaneous SCC in an 8-year old female Tortisehell cat. (**b**) Macroscopic image of a proliferative cutaneous SCC in a 10-year old male Tabby cat. (**c**) Macroscopic image of a cutaneous SCC in a 10-year old female cat (of unknown breed). The arrows point to the SCC lesion on each cat. It is interesting to note that all areas affected have white hair coat. Photos courtesy of Dr. Richard Malik (The University of Sydney).

Squamous cell carcinoma (SCC) is a malignant neoplasm of epidermal cells in the squamous epithelium. The most commonly occurring sites of SCC in cats are the skin and oral cavity. Cutaneous SCC (cSCC) accounts for 15% of skin tumours in cats, with most occurring on the head (Figure 4), often involving the pinna, eyelid and nasal planum (cSCC at the tip of the ears in white cars has a characteristic presentation) [61].

SCC is the most common malignant tumour of the oral cavity in cats (60–70% of all oral malignancies), with most involving the lingual region and dentate jaws [62,63]. Most SCCs are locally invasive and in particular, oral SCC (oSCC) can exhibit bone invasion and osteolysis. Tumour spread to regional lymph nodes may occur, but distant metastases are rare and usually do not occur until late in the disease process. Surgery is the most widely used treatment option, with the ability for complete excision dependent upon the location of the tumour and its size. For example, a retrospective study of 61 cats with cSCC (of the nasal planum or pinna) found that surgery provided a median disease-free time of 594 days [64]. In contrast, due to its location, rapid tumour progression and diagnosis often at a late stage, the median survival time of oSCC rarely exceeds 1 year [62,63,65].

Prolonged exposure to UV light, lack of skin pigment (Figure 4) and a sparse haircoat all contribute to the development of cSCC [66]. The mechanism frequently proposed for cutaneous SCC and its association with UV light involves mutation of the tumour suppressor gene p53. However, whilst a mutant form of p53 has been detected by immunohistochemistry in 13/19 (68%) feline cSCC cases (and 3/5 (60%) of feline oSCC cases) [67], genetic analysis of *TP53* has only been performed in feline oSCC cases to-date. A 1 base pair deletion/frameshift somatic mutation in exon 4 of *TP53* has been reported in one case of feline oSCC [58]. More recently, a group reported that 18/26 (69%) [68] and 21/31 (68%) [69] cases of feline oSCC carried non-synonymous mutations in exons 5–8. However, it is not clear whether these represent somatic or germline mutations as although these variants were only found at low frequency, or not at all, in normal mucosa, the normal samples were not from the same cat as the oSCC tumours. The same group also analysed the methylation status of 10 genes (*TERT*, *ZAP70*, *GP1BB*, *LRRTM1, FLI1*, *MiR124-1*, *MiR296*, *KIF1A*, *PARP15* and *MAGEC2*) in oSCC of cats, with statistically significant differences being found relative to normal and non-neoplastic mucosa from unaffected cats; *ZAP70*, *FLI1* and *MiR124-1* were hyper-methylated, whilst *LRRTM1*, *KIF1A* and *MAGEC2* were hypo-methylated [69]. Recently, the same group demonstrated that DNA obtained from oral brushing could be analysed for *TP53* mutation and DNA methylation (6-gene panel: *ZAP70*, *FLI1*, *MiR124-1*, *KIF1A*, *MAGEC2* and *MiR363*) status to differentiate feline oSCC from controls (with a 69% sensitivity and an 86% accuracy) [70]. This is a promising example of how understanding the genetics of feline cancers can aid with diagnostics.

The ability of papillomaviruses to cause disease in felines has long been recognized and there have been suggestions they may be a significant cause of feline SCC [71]. However, the causal relationship between papillomavirus and feline SCC is not well established and there are mixed reports of the relative proportion of SCC samples carrying papillomavirus. For example, a study using PCR primers to specifically amplify *Felis domesticus* papillomavirus-2 (FdPV-2) found papillomaviral DNA in 76% of UV-protected SCCs, and 42% of UV-exposed SCCs [72]. However, more recently a metagenomic approach using ‘ViroCap’ (a targeted capture and next generation sequencing tool to identify all known vertebrate DNA viruses) on 20 feline oSCC cases found that papillomavirus was not commonly associated with feline oSCC [73].

## 5. Soft Tissue Tumours

Soft tissue tumours (STT) is a generalised, and likely oversimplified, term for a diverse group of tumours that arise out of the extraskeletal connective tissues, such as fibrous connective tissue of the dermis or subcutis (fibrosarcoma), fat (liposarcoma), nerve sheaths (malignant nerve sheath tumours) and small blood vessels below the skin (perivascular wall tumours) [74]. In veterinary pathology, there is a drive towards more precise classification of these tumours, rather than lumping by general histologic characteristics, since in the dog these subtypes may have prognostic differences [75,76]. Malignant soft tissue tumours in the cat have historically and predominantly continue to be grouped as soft tissue sarcomas (STS) in the literature and account for approximately 7% of skin and subcutaneous tumours in cats, within which fibrosarcomas are common [6,15,77]. These tumours are often grouped together as they behave similarly; often presenting as pseudoencapsulated tumours that are locally invasive, with up to 20% chance of metastasis (if high-grade) and a high risk of recurrence after surgical excision due to the tumours being poorly demarcated [6,15,77,78]. Some other types of sarcomas, including histiocytic sarcoma, rhabdomyosarcoma and haemangiosarcoma, are typically much more aggressive with distinct histologic characteristics and thus are generally considered separately.

There is a subset of malignant soft tissue tumours that develop at injection sites, after administration of vaccines and drugs, or sometimes due to other causes of chronic localised inflammation (for example microchip implantation), which are termed feline injection-site sarcomas (FISS). Fibrosarcomas are a common histologic type of these tumours, however, osteosarcoma, chondrosarcoma and others have been reported [79]. These are typically aggressive, and despite surgical removal with wide margins and additional post-operative treatment, local recurrence is common and a main cause of euthanasia. It has been suggested the chronic inflammation that develops at the injection site plays an important role in the tumourigenic processes [80]. Indeed, the identification of the altered expression of matrix metalloprotease (MMP) family members in FISS compared to non-feline injection-site sarcomas and carcinomas (specifically levels of MMP2 and MT-MMP16, which were also significantly correlated with survival time) supports an underlying inflammatory pathogenesis for this tumour type [81]. In addition, it is well-established that inflammation can play key roles in driving tumour initiation, growth, progression and metastasis in human cancers (reviewed in [82]).

There have been very few studies looking at the underlying genetics of feline STS, with those to-date mostly focusing on the tumour suppressor gene *TP53*. A study of 150 domestic short-hair cats in the USA (50 with FISS and 100 ‘controls’ that were disease free at the time of the study) found a strong association with FISS and two specific germ line single nucleotide polymorphisms in *TP53* [83]. However, these findings were not replicated in a study using similar-sized cat population from Germany [84]. Somatic mutations in *TP53* have been reported in several studies of FISS, ranging in frequency from 24–60% of cases [85,86] and were found to be significantly associated with rapid tumour recurrence and reduced overall survival [86]. Somatic mutations in *TP53* have also been reported in other feline sarcomas, including spindle cell sarcoma, pleomorphic sarcoma and fibrosarcoma [56,87,88]. In contrast, no mutations were found in the coding regions of the tumour suppressor and cell cycle regulatory genes *p21^waf1/cip1^* and *p27^kip1^* in 45 feline fibrosarcomas [56].

Interestingly, microarray-based profiling of 46 feline sarcomas (19 FISS and 27 non-FISS) found numerous regions of highly recurrent copy number alterations, however, deletions of two specific regions were significantly associated with non-FISS cases, possibly suggesting a fundamental difference in the underlying genetics of FISS tumours from other STTs, or at the very least a discrete set of discriminatory markers [89]. The same group later used a higher resolution microarray platform to profile one of these FISS tumours and found DNA copy number imbalances involving several key cancer-associated genes including *TP53*, *KIT*, *PTEN*, *RB1* and *FAS* [90]. This study also found major alterations in chromosome structure, with complex intrachromosomal rearrangements typical of those seen in aggressive soft-tissue sarcomas of other species [90]. Similarly, a study using RNAseq to profile the transcriptomes of three FISS tumours with patient-matched normal tissue (as well as cultures of FISS-derived cell lines and feline primary skin fibroblasts) found many similarities at the gene expression level with that seen in STT of dogs and humans [91].

## 6. Mast Cell Tumours

**Figure 5:**
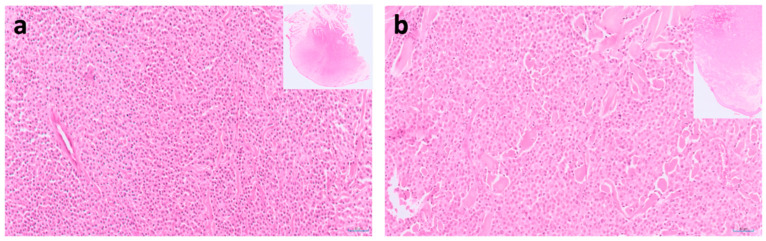
Two cases of feline cutaneous mast cell tumour. (**a**) Microscopic image of a well-differentiated mastocytic subtype of feline cutaneous mast cell tumour, in a 7-year old, male Burmese cat. The mitotic count was 3 per 10 HPFs (400×; 2.37 mm^2^). HE-stained, ×20 magnification (inset is low-power view). The long-term clinical outcome was reported as good, with a follow-up period of 1838 days. (**b**) Microscopic images of a well-differentiated mastocytic subtype of feline cutaneous mast cell tumour, in an 8-year old, male Domestic short hair cat. The mitotic count was 2 per 10 HPFs (400×; 2.37 mm^2^). HE-stained, ×20 magnification (inset is low-power view). The overall the survival time was 598 days, with clinical outcome reported as suspected metastatic spread (not histologically confirmed) involving the skin and the spleen. Critically, these cases show great similarity in histological appearance, but their outcomes were vastly different.

Mast cell tumours (MCTs) arise from mast cells, which are granulocytes that play a critical role in some immunological responses, and are found in a wide variety of tissues throughout the body. MCTs can occur in cats both as cutaneous (including subcutis) lesions and as visceral lesions (splenic and intestinal MCTs). The cutaneous manifestation is more common, with cutaneous MCTs (Figure 5) being the second most common type of skin cancer in cats, accounting for 15–21% of all feline cutaneous neoplasms [61]. MCTs are the most common cause of splenic disease in cats and frequently involve multiple other viscera and bone marrow, whereas gastrointestinal MCTs are rare (accounting for just 4% of all intestinal neoplasms in cats [92]). Although the clinical and histological features of MCTs are well described, the biological behaviour of these tumours is still an issue that is poorly understood (Figure 5), especially compared to dogs, which have well-established grading schemes with correlations to clinical outcomes available for cutaneous and subcutaneous MCTs [93,94,95,96]. Whilst many feline cutaneous MCTs may be benign, some can recur and/or spread to distant sites within months after excision [97].

MCTs of the gastrointestinal tract are generally viewed as an aggressive form of feline MCT with metastasis to mesenteric lymph nodes being common [98]. One study reported that cats with poorly differentiated intestinal MCTs survive 2–30 days compared to 28–538 days for those with well- or moderately differentiated tumours [99]. Thus, whilst surgical removal of MCTs may be curative for many low grade cutaneous MCTs, the prognosis is much more guarded when there is metastatic disease or involvement of the spleen or intestine.

There has been very little investigation of the genetics that underlie MCTs in felines. Indeed, to-date only the mutation status of *c-KIT* has been investigated, as it encodes the receptor tyrosine kinase Kit, which plays a key role in mast cell proliferation, differentiation and migration [100]. Activating mutations have been identified in exons 6, 8, 9 and 11 (Table 3) in feline MCTs, in both domestic and wild felines. *c-KIT* point mutations in exons 8 and 9 can cause ligand-independent homodimerisation and subsequent Kit autophosphorylation in MCTs from humans [101], canines [102] and felines [103]. In canine MCTs, *c-KIT* exon 11 mutations comprise 64–83% of all reported *c-KIT* mutations [102,104,105], and *c-KIT* mutations are associated with both reduced progression-free and overall survival rates [106]. Tyrosine kinase inhibitors (TKIs), such as imatinib mesylate, are routinely used in canine MCT and human mastocytosis patients to target mutant Kit [107]. However, whilst an in vitro study observed growth inhibitory effects for four different TKIs in three feline MCTs carrying exon 8 mutations in *c-KIT* [108] and in vivo studies have found a beneficial response to a variety of TKIs in ~70% of cats with MCTs at different sites [103,109,110], the prognostic relevance of *c-KIT* mutations in feline MCTs is questionable. Indeed, one study reported that the mutation status of *c-KIT* is not significantly related to Kit protein expression, is not strictly correlated with biological behaviour of the tumour and has no influence on prognosis [111].

## 7. Haemangiosarcoma

Haemangiosarcoma (HSA) is a malignant tumour, for which, at least in dogs, current evidence suggests it originates from a hematopoietic precursor cell rather than from the blood vessel lining (endothelial) cells [114,115], and as such it can occur anywhere in the body. However, evidence in the mouse suggests an endothelial precursor [116] and the authors could not find similar studies performed in the cat. The main types of HSA in cats are dermal/cutaneous, subcutaneous/intramuscular and visceral (involving internal organs) [117]. The most common forms of HSA in cats are dermal/cutaneous and subcutaneous, which tend to form on the head [117], suggesting that sun exposure may be a risk factor, as it is for angiosarcoma (AS) of the head/face/neck/scalp (HFNS) in humans [118]. This is in contrast to dogs, in which visceral forms, particularly the splenic and right atrial/auricular, are more common [15]. Surgical removal of dermal/cutaneous HSAs in cats tends to be curative, with the subcutaneous forms tending to recur after surgery due to the difficulty for complete excision [117]. Surgical resection of visceral tumours may be possible for localized disease, however, as in dogs, metastatic disease is commonly already present at the time of diagnosis in cats [15,119].

There is only one study that has looked at the genetics of HSA in cats. A targeted sequencing approach was taken to look at ~1000 cancer-associated genes in paired tumour-normal samples of 13 feline cutaneous HSA cases [120]. The most recurrently mutated genes in the HSA samples were *TP53* (6/13, 46% cases) and *NOTCH1* (2/13, 15% cases) [120]. It is interesting to note that in human HFNS AS, the most commonly mutated gene is *TP53* (9/19 patients, 47% cases), with *NOTCH1* also being frequently mutated (5/19 samples, 26% cases) [120]. In addition, there were mutations in other genes in the feline HSA samples that have been shown to be recurrently mutated in human AS samples, including *ATRX*, *GLI1*, *MTOR*, *PCLO*, *PGR*, *PIK3CA*, *RELN*, *SETD2* and *TERT* [120]. Copy number analysis of the feline cutaneous HSA samples showed relatively few somatic copy number alterations compared to human AS, with the most penetrant SCNAs being in smaller regions of chromosomes A2 and D2 [120]. In terms of these putative pathogenic germline variants in the feline orthologs of established human AS susceptibility genes, the same study found missense mutations in *ERCC2*, *RB1*, *IDH1*, *IDH2*, *POT1*, *TP53* and *XPC* [120]. However, further analysis of larger cohorts will be needed to determine whether these genes/alleles do indeed play a role in germline predisposition to cutaneous HSA in cats.

## 8. Pulmonary Carcinoma

**Figure 6:**
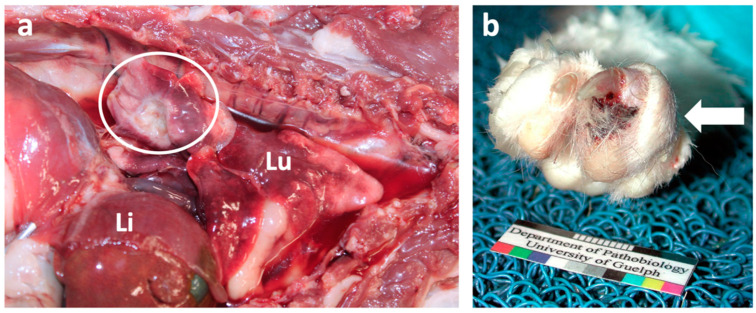
**Macroscopic images of a pulmonary carcinoma and digital metastases in a cat.** (**a**) Postmortem examination of a 12-year old female Domestic short hair cat with a focal pulmonary carcinoma (circle) in the right caudal lobe (Lu) and pleural effusion. The liver (Li) is marked for orientation. (**b**) Postmortem examination of a female Domestic short hair cat (of unknown age) with a digital mass (arrow), confirmed to be a metastasis from a pulmonary carcinoma. Photos courtesy of Dr. Jeff Caswell (Department of Pathobiology, University of Guelph).

Primary pulmonary carcinoma is rare in cats, with one study finding they represented only 0.69% of all feline cases admitted to a veterinary teaching hospital over a 4.5 year period [121]. It is an aggressive neoplasm with one study finding that regardless of the histological subtype, ~80% of the feline pulmonary carcinomas had metastasized at the time of diagnosis (Figure 6a), with the most frequent type of metastasis being intrapulmonary metastasis (66.7%) [121]. Treatment frequently involves lung lobectomy and adjuvant chemotherapy, and depending on the degree of differentiation of the tumour, the median survival time can be as much as 698 days or as low as 75 days [122]. However, a significant number of feline lung tumours are classified as inoperable at diagnosis, due to extensive disease, metastasis or concurrent decompensated cardiomyopathy [123]. Additionally, some cats may present clinically for lameness due to digital metastases and be subclinical for the primary pulmonary carcinoma [124], so called “lung-digit syndrome” (Figure 6b).

There have only been three studies that have investigated the genetics of lung cancer in cats, and all of them have focused on the genes known to be important in human lung cancer, specifically *TP53* and *K-RAS* [125] and *HER2* (in a subset of human lung cancers) [126]. Specifically, a feline bronchioloalveolar carcinoma cell line (SPARKY) was demonstrated to have a mutation in *TP53* at codon 167, but no alterations were seen in *K-RAS* (or *H-RAS*), and the karyotype was aneuploid (with evidence of genomic inability) [127]. However, sequencing of key regions of *K-RAS* and *TP53* in three feline lung carcinomas (and matched normal lung) did not reveal any alterations [121]. More recently, fluorescence in situ hybridisation (FISH) in a dual-core tissue microarray was used to demonstrate *HER2* amplification in 3/13 cases of feline pulmonary carcinoma, which significantly associated with HER2 overexpression as analysed by immunohistochemistry [128].

## 9. Pancreatic Carcinoma

Pancreatic carcinoma in cats most commonly involves the exocrine acinar cells of the pancreas and it is an aggressive disease that has frequently undergone distant metastasis at the time of diagnosis [129]. As such, pancreatic carcinoma in cats is associated with a poor prognosis; the survival time is 2–4 months, with diagnosis frequently occurring at necropsy [15,129]. Longer survival times have been reported in cats who have their masses surgically removed (median survival time >300 days) [130] or cats treated with chemotherapy (toceranib phosphate treatment resulted in a survival time of 792 days post-diagnosis) [131].

As *KRAS* mutations occur at a high frequency in human ductal pancreatic carcinomas (although rarely in pancreatic acinar cell carcinomas), all studies on pancreatic carcinoma in felines to date have focused on the mutational status of *KRAS* (in particular codon 12, which is a hotspot in human cancers) [132]. One study found codon 12 *KRAS* mutations in 2/3 cats [133], although it is not clear whether these cases were acinar or ductal origin. A subsequent study of 18 cats with pancreatic acinar cell carcinoma found no *KRAS* mutations in either codon 12 or 13 [134], similar to that seen in pancreatic acinar cell carcinoma in humans.

## 10. Osteosarcoma

Osteosarcoma (OSA) is a malignant neoplasm arising from bone and is histologically characterised by the presence of tumour-derived osteoid. Primary bone tumours in cats are rare (an incidence of 4.9 of 100,000 individuals), with the majority of these being osteosarcoma (70–80%) [135,136]. They are characterised based on their location in and around the bone, with central (medullary) OSAs being the most common in dogs and cats. OSAs can arise from any bone, but are slightly more common from appendicular skeletal sites than axial ones [135]. Prognosis is dependent upon the location of the tumour, with axial OSA generally being poor (with an average survival time of 6 months) in contrast to appendicular OSA, which is associated with a more favourable outcome, particularly when treated with complete surgical excision or amputation (with an average survival time of 26–49 months) [137]. Although historically considered to have a better prognosis compared to dogs, a more recent report suggests a shorter median survival time and metastatic rate than previously reported, with a median survival time of 527 days after limb amputation and distant metastases in 46.3% of all cases [138].

To-date there has only been one investigation into the genetics of OSA in cats and that was identification of a somatic missense mutation in codon 273 of exon 8 of *TP53* in an OSA from the shoulder of an 8-year old female cat [55]. This is interesting as this codon is a hotspot in human cancers. Unfortunately, overall feline OSA has not garnered the same research attention as canine OSA.

## 11. Looking towards a Future of Greater Understanding of the Feline Oncogenome

The recent release of a high-quality reference genome for the domestic cat [14] heralds the start of new and exciting times for our ability to understand the feline oncogenome. For example, alignment of whole-genome sequencing data from 54 domestic cats to the newly released reference genome, identified genome-wide sequence variant information for this species, specifically single nucleotide variants (SNVs) and structural variants (SVs) [14]. Of the 16 loss-of-function SNVs that were identified, the most notable was that of a potential cause for early onset feline mediastinal lymphoma, with a stop gain in tumour suppressor gene *FBXW7* (found in one cat and its offspring) [14]. Screening of additional cats will be needed to validate this *FBWX7* stop gain as a causative mutation for mediastinal lymphoma susceptibility, however it is an exciting prospect that we may be close to identifying a candidate gene/mutation for inherited cancer susceptibility in cats, and serves to show the benefits that are to be gained from now having a high-quality reference genome for the domestic cat. These are truly exciting times for feline oncogenomics and it is hoped that this will rapidly translate into significant advances in diagnosis, prognosis and treatment of cancer in cats.

## Figures and Tables

**Table 1:** A summary of studies looking at the type of tumours most commonly found in domestic feline populations and their relative severity of the tumour.

Population	Benign Versus Malignant	Study
59 tumours from 56 domestic cats in Tulsa, USA (1972 to 1973)	Overall, 83% of the tumours were malignant	[4]
18,375 tumours from 51,322 domestic cats in Switzerland (1965–2008)	Overall, 80% of the tumours were malignant	[5]
9683 cutaneous tumours from 9200 domestic cats in the UK (2006–2013)	Overall, 53% of the tumours were malignant	[6]
685 tumours from domestic cats in Mexico City, Mexico (2006–2018)	Overall, 85% of the tumours were malignant	[7]
475 tumours from 417 domestic cats in Portugal (2019)	Overall, 75% of the tumours were malignant	[8]
1724 tumours from domestic cats in Portugal (2019–2020)	Overall, 75% of the tumours were malignant	[9]

**Table 2:** Examples of feline lymphoma cell lines and how they have been used to understand the genetics of feline lymphoma.

Lymphoma Cell Lines: Genetic Investigations Performed and Results Obtained
3201 cell line [26]:Examined for mutations in *TP53*; a non-synonymous mutation was found at codon 235
3201, FT-1, FL-74, KO-1, R96 cell lines [27]:Cytogenetic analysis and examined for mutations in *TP53* and mRNA expression levels of *MDM2*; 3201, FL-74 and R96 showed centrosomal amplification and chromosomal instability, mutations in *TP53* were found in all the cell lines (although only 3201 had a non-synonymous mutation at codon 235), and none of the cell lines showed elevated *MDM2* mRNA levels
FL-7, FT-1, 2301, KO-1, R96 cell lines [28]:Examined for mutations in *GADD45*; no mutations were found
S87 cell line [29]:Cytogenetic analysis; showed centrosomal amplification and chromosomal instability
3281, FT-1, MS4 cell lines [30]:Treated with a DNA methylation inhibitor; aberrant gene expression patterns observed
FT-1, MS4, KO-1 cell lines [31]:Genome-wide methylation profiling; showed thousands of CpG sites with gain of methylation at normally unmethylated CpG islands and loss of methylation at normally methylated non-CpG islands

**Table 3:** **A summary of the studies that have analysed the *c-KIT* gene in feline MCTs.** The Kit protein is composed of an extracellular domain, consisting of 5 immunoglobulin-like domains (encoded by exons 1–9), a transmembrane domain (exon 10) and an intracellular domain (exons 11–21), which contains a negative regulatory juxtamembrane domain (exons 11–12) and a cytoplasmic tyrosine kinase domain (exon 13 and 17).

Samples	*c-KIT* Mutations Reported	Study
20 cutaneous MCTs from domestic cats 4 cutaneous MCTs from cheetahs	• Domestic cat cases: non-synonymous mutations found in exons 6, 8 or 9 in 60% of cases (none in exon 11). One lesion harboured 2 independent exon 6 mutations. • Cheetah cases: 2/4 showed mutations in exon 6 (but either synonymous or conserved amino acid change). No mutations found in other exons.	[112]
16 intestinal MCTs from domestic cats	• No mutations found.	[99]
24 cutaneous MCTs from domestic cats	• Mutations present in 19 (56%) tumours. • Exon 8 (19% cats), exon 9 (71% cats), exon 11 (10% cats). • In 6 of 9 (67%) cases, multiple nodules from the same cat had different mutational statuses.	[111]
62 cutaneous, splenic or widespread MCTs from domestic cats	• Mutations present in 42 (67%) tumours. • Exon 6 (1/17 cats), exon 8 (28/62 cats), exon 9 (15/62 cats), exon 11 (1/62 cats), exon 13 or 17 (0/62 cats) • 3 cats carried mutations in both exon 8 and 9	[103]
10 splenic MCTs from domestic cats	• No mutations were found (looked at exons 11, 12 and 17).	[113]

## Data Availability

Not applicable.
